# Comparative Analysis of Spacer Targets in CRISPR-Cas Systems of Starter Cultures

**DOI:** 10.32607/actanaturae.27533

**Published:** 2024

**Authors:** A. A. Fatkulin, T. A. Chuksina, N. P. Sorokina, I. T. Smykov, E. V. Kuraeva, E. S. Masezhnaya, K. A. Smagina, M. Yu. Shkurnikov

**Affiliations:** Higher School of Economics, Faculty of Biology and Biotechnology, Moscow, 101000 Russian Federation; Gorbatov Federal Research Center for Food Systems, Moscow, 109316 Russian Federation

**Keywords:** bacteriophage, CRISPR-Cas systems, cheesemaking, starter cultures, One Health

## Abstract

Dairy production facilities represent a unique ecological niche for bacteriophages of lactic acid bacteria. Throughout evolution, bacteria have developed a wide range of defense mechanisms against viral infections caused by bacteriophages. The CRISPR-Cas system is of particular interest due to its adaptive nature. It allows bacteria to acquire and maintain specific resistance to certain bacteriophages. In this study, we investigated the CRISPR-Cas systems of lactic acid bacteria. Special attention was paid to the specificity of the spacers in CRISPR cassettes. CRISPR-Cas systems were found in the genomes of 43% of the lactic acid bacteria studied. Additionally, only 13.1% of the total number of CRISPR cassette spacers matched bacteriophage genomes, indicating that many predicted spacers either lack known phage targets or are directed against other types of mobile genetic elements, such as plasmids.

## INTRODUCTION

In the production of fermented dairy products, starter cultures are used to promote milk fermentation and to form a product with distinctive textural, aromatic, and flavor properties [1]. However, the lactic acid bacteria used in this process can be susceptible to bacteriophage infection [2], as dairy production facilities represent a unique ecological niche for bacteriophages of lactic acid bacteria, which are present in raw milk [3].

Throughout evolution, bacteria have developed a wide range of defense mechanisms aimed at protecting themselves against viral infections caused by bacteriophages. These mechanisms include, among others, abortive infection (Abi) systems, restriction-modification (R-M) systems, and CRISPR-Cas systems [4]. The particular interest in CRISPR-Cas systems is due to their adaptive nature, which allows bacteria to acquire and maintain specific resistance to certain bacteriophages [5]. CRISPR-Cas-mediated immunity is found in approximately half of sequenced bacteria and in most archaea [6], making it one of the key elements of antiviral defense in prokaryotes.

Currently, two classes of CRISPR-Cas systems are recognized, consisting of six types (I–VI) which differ in their mechanisms of action and constituent elements [6]. Despite this diversity, all CRISPR-Cas systems share a number of characteristic features. The main element of each CRISPR-Cas system is the CRISPR locus. It contains CRISPR-associated (cas) genes that are responsible for interacting with foreign nucleic acids, as well as a CRISPR cassette: short palindromic repeat sequences of DNA separated by unique insertions — spacers. The spacers are fragments of foreign DNA integrated into the bacterial genome as a result of a previous infection [5]. They determine the sequence that will be recognized by Cas nucleases and, consequently, play a key role in CRISPR-Cas immunity. Most spacers are relatively short: for example, it is known that for the I-E and I-F subtypes, spacer lengths range from 31 to 33 bp, while for I-B, I-C, I-D, and I-U, they range from 34 to 37 bp [7].

The mechanism of action of the CRISPR-Cas system can be divided into several key stages: when foreign nucleic acid enters the bacterial cell, new spacers are integrated into the CRISPR cassette. This is followed by the transcription of the spacers, leading to the formation of precursor CRISPR-RNAs (pre-crRNA), which are then processed into mature crRNAs. These crRNAs, binding with Cas nucleases, form an active complex capable of recognizing and binding to the complementary sequence of foreign DNA or RNA. Upon target binding, the foreign genetic material is degraded, providing protection to the cell from repeated infections [5]. For successful degradation of the bacteriophage genome, the target region must have a high degree of homology with the spacer. It has been previously shown, for instance, that the presence of three or more mutations can lead to an almost complete inactivation of CRISPR-Cas immunity [8].

In this study, the CRISPR-Cas systems of lactic acid bacteria were investigated. Special attention was paid to the specificity of the CRISPR cassette spacers, which allowed for an assessment of these bacteria’s resistance to known bacteriophages.

## EXPERIMENTAL

The genome sequences of lactic acid bacteria, as well as bacteriophages from the *Caudoviricetes *class, were obtained from the NCBI database. The genomic data were preprocessed to remove duplicates. The PADLOC tool [9] was used to identify CRISPR-Cas systems in bacterial genomes. MinCED [10] was used to predict spacers in the bacterial genomes, after which they were aligned to the phage genome sequences using Bowtie2 [11], applying the “–ndto- end” option and the “–ery-sensitive” preset. To establish the functions of the regions to which the spacers were aligned, the bacteriophage genomes were further annotated using Pharokka [12]. To assess the overrepresentation of functional groups among the spacer targets, the proportion of spacers aligned to genes in each group and the proportion of genes in each group relative to the total were calculated. Then, to determine whether the distribution of spacers across groups was uniform, a Fisher’s exact test following the “one-vs-all” principle was applied.

## RESULTS

**Fig. 1:**
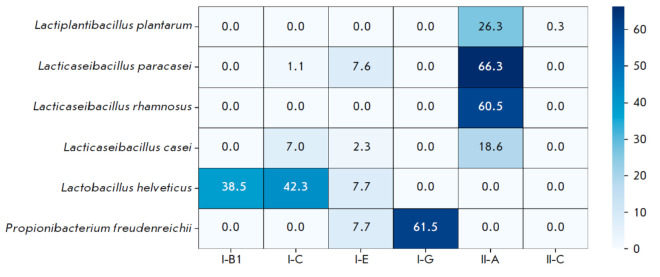
Proportion (%) of CRISPR-Cas Systems in the Genomes of LAB

A total of 563 genomes of lactic acid bacteria, belonging to 6 species, were obtained from the NCBI database (*Table 1*). Using PADLOC, CRISPR-Cas systems were identified in 243 of these genomes (*Table 1*), corresponding to approximately 43% of all the genomes studied. The predicted CRISPR-Cas systems belong to 6 different subtypes: I-B, I-C, I-E, I-G, II-A, and II-C (*Fig. 1*). In the genomes of *Lactiplantibacillus plantarum*,* Lacticaseibacillus paracasei*, and *Lacticaseibacillus rhamnosus*, subtype II-A systems dominate, which may be an indirect indication of their important role in the defense mechanisms of these species. Among the strains of *Lacticaseibacillus casei*, the subtypes I-C and II-A are prevalent, while for *Lactobacillus helveticus*, the subtypes I-B and I-C are characteristic. In the genomes of *Propionibacterium freudenreichii*, subtype I-G predominates.

**Table 1:** Distribution of CRISPR-Cas Systems in the Genomes of Lactic Acid Bacteria

Species Name	Number of Genomes	Contains CRISPR-Cas, %
Lactiplantibacillus plantarum	300	80 (26.7)
Lacticaseibacillus paracasei	92	68 (73.9)
Lacticaseibacillus rhamnosus	76	46 (60.5)
Lacticaseibacillus casei	43	10 (23.3)
Lactobacillus helveticus	26	22 (84.6)
Propionibacterium freudenreichii	26	17 (65.4)
	563	243

**Fig. 2:**
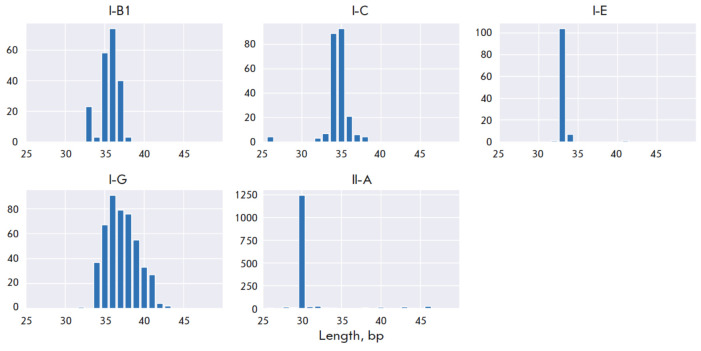
Distribution of Predicted Spacer Lengths by CRISPR-Cas Subtypes

The total number of spacers predicted using MinCED in the bacterial genomes amounted to 6,971 (*Table 2*); however, many sequences are overrepresented within species. For this reason, the number of unique spacers among the studied species was only 3,477. The distribution of the lengths of the predicted spacers (*Fig. 2*) is consistent with previously published data [7]. Subsequent alignment to the genomes of 21,261 phages of the *Caudoviricetes *class, obtained from the NCBI database, yielded 916 matches (*Table 2*), of which only 485 are unique in terms of sequence and species origin.

**Table 2:** Distribution of Spacers in the Genomes of LAB

Species Name	Total Predicted	Identified in Phages, %
Lactiplantibacillus plantarum	1519	67 (4.4)
Lacticaseibacillus paracasei	2128	296 (13.9)
Lacticaseibacillus rhamnosus	1239	289 (23.3)
Lacticaseibacillus casei	379	53 (14.0)
Lactobacillus helveticus	778	48 (6.2)
Propionibacterium freudenreichii	928	163 (17.6)

**Fig. 3:**
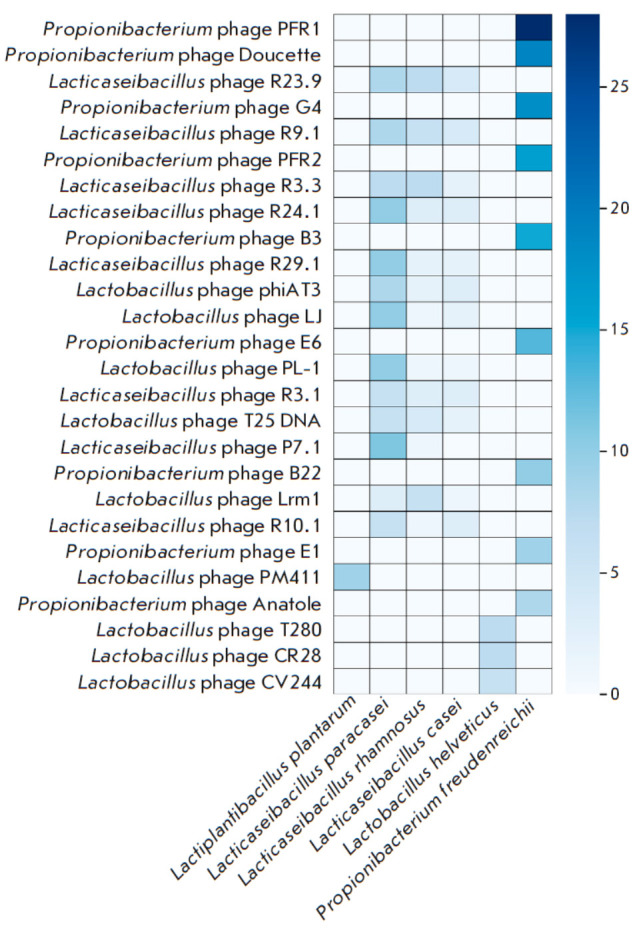
Alignments of Unique Spacers to Bacteriophage Genomes. Phages with fewer than 5 spacers aligned from each species were excluded for better visual clarity

All the obtained alignments correspond to the genomes of 69 phages, which were previously described as bacteriophages of lactic acid bacteria (*Fig. 3*). Functional annotation of the phage genomes revealed that the predicted spacers more frequently aligned to the genes encoding tail proteins, the genes involved in packaging, and the genes participating in DNA metabolism (adjusted *p*-value < 0.05) (*Table 3*).

**Table 3:** Genes Overrepresented Among Spacer Targets

Gene (according to Pharokka annotation)	Adjusted p-value
Terminase small subunit	3.8 × 10-6
Head-tail adaptor Ad1	2.5 × 10-3
Head scaffolding protein	3.6 × 10-3
Major tail protein	3.8 × 10-3
DNA repair exonuclease	3.9 × 10-2

## DISCUSSION

The study revealed the presence of CRISPR-Cas systems in 43% of the investigated lactic acid bacterial genomes, confirming their significant role in the defense mechanisms of these microorganisms against foreign nucleic acids, including bacteriophage genetic material. The results also demonstrate the diversity of CRISPR-Cas systems across different species of lactic acid bacteria.

The relatively low percentage of spacers matching bacteriophage genomes (only 13.1% of the total) may indicate that many of the predicted spacers either do not have known phage targets or are directed against other types of mobile genetic elements, such as plasmids. This observation also highlights the need for further research to deepen our understanding of the interactions between CRISPR-Cas systems and various mobile genetic elements. Additionally, the discovery and description of new, previously unknown bacteriophages remains a relevant area of study.

Notably, among the spacer targets, genes responsible for viral particle packaging, tail protein genes, and genes involved in DNA metabolism are overrepresented, as these regions are likely to be more conserved due to their functions related to key stages of the viral life cycle, such as virion assembly and entry into the host cell.

It is also noteworthy that the phage spectra to which *Lacticaseibacillus paracasei*, *Lacticaseibacillus rhamnosus*, and *Lacticaseibacillus casei *strains are resistant display a clear similarity. This fact, combined with the similarity of the CRISPR-Cas system subtypes found in the genomes of these strains, may suggest common defense mechanisms or indicate that these bacteria have followed similar evolutionary paths in developing resistance to bacteriophages.

In this study, we conducted a comprehensive analysis of CRISPR-Cas systems found in the genomes of lactic acid bacteria. The results largely align with previously published data; however, in our work, we used the most up-to-date information sources and focused on studying CRISPR-Cas-mediated immunity in several strains. Additionally, we examined the specificity of the identified spacers in more detail, including investigating the functions of the regions they target. Thus, the results described in this article not only broaden the current understanding of the role of CRISPR-Cas in the adaptive immunity of lactic acid bacteria, but also underscore the importance of further research in this area.

## CONCLUSION

Further research is needed to better understand the role of the CRISPR-Cas system in protecting starter cultures from bacteriophages and to evaluate its impact on the fermentation process. The abundance of bacteriophages infecting starter cultures in dairy facilities highlights the importance of analyzing the resistance spectrum of starter cultures for their rational combination, depending on the phage spectrum in raw milk.

## Abbreviations

R-M: restriction-modification system
Abi: abortive infection system
